# The Potential Utility of Pharmacogenetic Testing in Psychiatry

**DOI:** 10.1155/2014/730956

**Published:** 2014-12-17

**Authors:** Kathryn R. Gardner, Francis X. Brennan, Rachel Scott, Jay Lombard

**Affiliations:** Genomind, Inc., 2200 Renaissance Boulevard, Suite 100, King of Prussia, PA 19406, USA

## Abstract

Over the last decade, pharmacogenetics has become increasingly significant to clinical practice. Psychiatric patients, in particular, may benefit from pharmacogenetic testing as many of the psychotropic medications prescribed in practice lead to varied response rates and a wide range of side effects. The use of pharmacogenetic testing can help tailor psychotropic treatment and inform personalized treatment plans with the highest likelihood of success. Recently, many studies have been published demonstrating improved patient outcomes and decreased healthcare costs for psychiatric patients who utilize genetic testing. This review will describe evidence supporting the clinical utility of genetic testing in psychiatry, present several case studies to demonstrate use in everyday practice, and explore current patient and clinician opinions of genetic testing.

## 1. Introduction

Mental illnesses are extremely prevalent and debilitating. Depression alone is the leading cause of disability worldwide, leading to a significant patient/economic burden, affecting at least 350 million people [1]. Approximately 14% of the global disease burden can be attributed to neuropsychiatric disorders [2]. Twenty-five percent of adults in the US currently suffer from a mental illness, and at least half will develop one or more in their lifetime [3]. Moreover, 50% of patients suffering from depression do not respond to first-line therapies or experience severe adverse reactions to medications [4].

There is significant interindividual variation to psychotropic treatment response, leading psychiatrists to adopt a trial and error approach to treatment [5]. Genetic variability can account for much of this inconsistency in medication response [6]. Knowledge of a patient's genetic background can help clinicians provide a personalized medicine strategy by predicting both drug response and risk for adverse events [7]. Clinicians can utilize this information to compensate for a gene defect (pharmacodynamic genetic variations) or to adjust medication dosage to accommodate the rate at which the patient metabolizes different medications (pharmacokinetic genetic variations).

Much of the utility of pharmacogenetic testing has been shown in clinical settings other than psychiatry. Many of these tests identify mutations relating to altered expression and functions of genes associated with drug disposition and response and have been useful in clinical practice [8]. Within psychiatry, several studies have found genetic variations associated with altered treatment response/efficacy [9, 10] and increased side effect risk [11–15]. Genetic testing for such variations can help identify which patients are more or less likely to respond to psychotropics and which are likely to experience an increased side effect burden. Incorporation of this information can drive appropriate treatment choices to improve treatment outcomes [16].

## 2. Clinical Utility

Understanding the utility (i.e., the ability to improve patient outcomes) of genetic tests applied in one field can facilitate adoption in areas where testing is not presently employed. For example, a genetic test currently used in medical practice analyzes genetic variations in thiopurine methyltransferase (*TPMT*). This enzyme is necessary for proper thiopurine treatment in patients suffering from acute lymphoblastic leukemia. Patients who express a defective version of this enzyme can experience life-threatening adverse events in response to treatment [8].* TPMT* testing allows for individualization of therapy and has been shown to be cost effective in patients who are treated with azathioprine [8]. Similarly, in psychiatry, variations in the serotonin transporter protein (*SLC6A4*) have been found to help predict how patients will respond to antidepressant treatment. As* SLC6A4* is the primary target for selective serotonin reuptake inhibitors (SSRIs), patients with a variation in this protein may show poor response, lower remission rates, and increased side effects leading to medication intolerance with SSRIs [22].

Methylenetetrahydrofolate reductase (*MTHFR*) is another gene regularly tested in the field of cancer biology and has growing implications within psychiatry. Patients with variations in* MTHFR*, particularly the C677T variation, have decreased enzyme activity and variable folate levels [8]. In the review by Dervieux et al., the* MTHFR* variant depicts a risk for increased side effects in response to methotrexate therapy, a folic acid antagonist [8]. Methotrexate is a drug used to treat cancer and for immunosuppressive therapy, but serious and life-threatening side effects are associated with its use [8]. Genetic testing for* MTHFR* variations has been shown to effectively predict which patients are more likely to suffer from these serious adverse events in response to methotrexate treatment [8].

In addition to its role in methotrexate response, MTHFR is also a necessary enzyme in the pathway to produce methylfolate and ultimately monoamine neurotransmitters associated with mood regulation [23]. Deficiencies of methylfolate have been implicated in neurological disorders [8]. As the C677T variation has been shown in many different settings to lead to decreased enzyme activity of MTHFR, it is reasonable that disruption of this enzyme would also impact methylfolate levels, neurotransmitters, and depression [23]. L-methylfolate has been found to be an effective augmentation strategy for patients who show little or no response to SSRI/SNRI treatment, as well as helping to improve patient adherence to these medications [24, 25]. Additional data showed that patients who are homozygous or heterozygous for the risk allele at C677T have a greater improvement in their Hamilton Depression Rating Scale scores after taking adjuvant L-methylfolate compared to placebo [26]. Genetic testing for this variation in psychiatry can help clinicians determine an effective treatment strategy for those with compromised ability to activate folate.

While still evolving, the use of pharmacogenetic testing in psychiatry is expected to become widespread [27]. A recent meta-analysis examined 294 previously published papers regarding the efficacy and utility of pharmacogenetic testing for several genes related to psychiatric treatment outcomes [10]. Fifty-seven percent of the papers examined demonstrated significant associations between genetic variations and improved patient outcomes [10]. Clinical response and remission were significantly associated with variants within* SLC6A4* and cytochrome P450 2D6 (*CYP2D6*), as well as serotonin receptor 2A (*5HTR2A*) and cytochrome P450 1A2 (*CYP1A2*) [10]. Adverse events were most associated with variations in* CYP2D6*, serotonin receptor subtype 2C (*5HT2C*),* SLC6A4,* and* 5HTR2A* [10]. These examples advocate the usefulness of genetic testing in psychiatric clinical practice and highlight the need for continued development of new genetic tests which identify variations associated with altered treatment response and efficacy [8].

## 3. Cost Effectiveness

In addition to improved treatment outcomes, genetic testing can reduce high costs related to treatment failures and severe adverse events [8]. The total medical expenditure for mental illnesses in the US was $83.6 billion dollars in 2012 and is only expected to increase [3]. Individuals with treatment resistant depression have an even higher cost burden, with 29% to 40% higher medical costs [28, 29]. Several studies have examined the clinical utility and cost effectiveness of pharmacogenetic testing in psychiatry, demonstrating positive findings (Table 1). A systematic review of 20 studies, including genetic screening tests for cytochrome P450 enzymes, found that most economic analyses reported genetic testing to be cost effective [7]. Several additional studies evaluated cost and treatment outcomes in psychiatric patients with altered cytochrome P450 enzyme metabolism and also demonstrated cost savings [17–19]. Increased costs of $4,000 to $6,000 were found for patients with severe mental illnesses who have either poor or ultrarapid metabolism of the CYP2D6 [18]. In addition, longer hospital stay durations were identified in patients with major depressive disorder categorized with a severe mental illness and who exhibit CYP2D6 poor metabolism [18, 19].

A retrospective study examining antidepressant response based on variations in cytochrome P450 enzymes (*CYP2D6*,* CYP2C19*,* CYP2C9*, and* CYP1A2*),* SLC6A4,* and* 5HTR2A* found individuals with genetic variations relating to adverse clinical outcomes had 69% higher total healthcare costs, 67% more general medical visits, 3-fold higher medical absence days, and 4-fold greater number of disability claims than individuals without the associated risk variations [20]. Additionally, pharmacogenetic testing for variants in* CYP2D6* and* CYP2C19* leading to poor or ultrarapid metabolism was found to reduce overall medical costs by 28% in schizophrenic patients [17]. A recent study also demonstrated patients who receive genetic testing, with the Genecept Assay (Genomind, Inc.), had reduced costs as compared to matched controls [21]. Given findings that demonstrate a single antidepressant treatment failure result in increased costs of $1,043 in the first postepisode year [30], it is not surprising that genetic testing has begun to demonstrate dramatic cost savings for psychiatric patients.

Moreover, reductions in healthcare costs with pharmacogenetic testing can be accompanied by increased medication adherence. Medication adherence is a problem, spanning many areas of mental illness including 31% of patients with schizophrenia or schizoaffective disorders, 33% of patients with bipolar disorder, and 41% of patients with other severe mental illnesses [31]. Several studies have shown that genetic variations can lead to increased side effect risk and medication intolerability, which result in higher levels of medication discontinuation and noncompliance [13, 32], as well as higher overall medical costs [18, 20]. As an example, the short (S) allele in the serotonin-transporter-linked polymorphic region (5HTTLPR) of the promoter region of* SLC6A4* has been associated with decreased adherence due to side effects [32]. Additionally, a study examining CYP2D6 metabolizer phenotypes in schizophrenic patients found that poor metabolizers were more likely to experience tardive dyskinesia and extrapyramidal symptoms and had a significantly higher prevalence of noncompliance compared to intermediate or extensive metabolizers [13]. In particular, patients who receive genetic testing have been shown to have increased adherence to medications [21]. These data suggest that genetic testing can allow clinicians to determine which patients are likely to suffer from adverse effects and medication intolerability and provide them with alternative treatment plans resulting in improved patient adherence and lower healthcare costs.

An example of one commercially available genetic test for psychiatric patients is the Genecept Assay. This assay examines ten genes associated with treatment response, side effects, metabolism, tolerability, and overall efficacy of many psychiatric medications [11, 14, 33–35]. The assay analyzes variations in three cytochrome P450 pharmacokinetic genes,* CYP2D6*,* CYP2C19*, and* CYP3A4*, and seven pharmacodynamic genes,* SLC6A4*,* 5HT2C*, a calcium channel subunit (*CACNA1C*), a dopamine receptor subtype (*DRD2*), catechol-O-methyl transferase (*COMT*), ankyrin G (*ANK3*), and* MTHFR*. A study was conducted analyzing the cost effectiveness and impact on medication adherence in psychiatric patients who received this genetic test compared to a matched set of controls [21]. This study utilized healthcare claims data and pharmacy claims data and found that patients who utilized the assay saved an average of $562 over a four-month span, relative outpatient cost savings of 9.5% [21]. This finding demonstrates a conservative estimate of the total cost savings as inpatient costs could not be measured [21]; it is possible that if this variable was taken into account, an even greater cost decrease would have been observed. In addition to significant cost savings, these patients demonstrated a 6.3% increase in medication adherence, compared to controls who only showed a 0.3% increase in adherence [21]. These findings, along with the data previously described, demonstrate the clinical utility and cost effectiveness of pharmacogenetic, testing and, in particular, its importance, and relevance to improving psychiatric care.

## 4. Pharmacogenetic Testing in Everyday Psychiatric Practice

Armed with patient genetic information, clinicians can more quickly identify effective therapies, thus limiting the prolonged suffering and economic burden placed upon many patients with chronic illnesses. Several examples are available which demonstrate the utility and effectiveness of testing in practice for a variety of clinical diagnoses. This is demonstrated in the following published patient cases. One case describes an 18-year-old male diagnosed with intermittent explosive disorder, suffering from uncontrolled anger outbursts and several failed medication trials, who elected to utilize genetic testing [36]. The results guided his clinician to initiate lithium, which markedly reduced his symptoms, with no adverse medication effects, and allowed him to improve his school work, social, and family life [36]. Lithium was chosen as an intervention for this patient as he had variations in the* SLC6A4*,* DRD2*, and* 5HT2C*, increasing his risk for failure and intolerance with SSRIs and antipsychotic agents. Thus, a treatment strategy which did not target the serotonin transporter or dopamine receptor pathway would likely be better tolerated in this patient [36]. In another patient case, a clinician treating a 31-year-old female suffering from severe depressive symptoms was able to utilize genetic testing, and the resulting therapeutic choices led to complete remission of the patient's symptoms [37]. In this example, lamotrigine was chosen in response to clinical presentation, as well as a variation in the* ANK3* gene [37]. ANK3 is a protein related to sodium channels and is involved in neuronal excitability [38]. Lamotrigine was utilized to stabilize the patient's mood and for its potential as a modulator of sodium channel activity [37, 39].

Genetic testing not only helps identify conventional treatments which may be most effective, but also can help identify effective alternative therapeutic options. In this last case, a clinician utilized genetic testing for a 69-year-old man suffering from long-term depression symptoms [40]. This patient had a variation in* MTHFR* which led the clinician to prescribe L-methylfolate, leading to complete remission of symptoms [40]. As described previously, variations in the* MTHFR* gene may lead to impaired neurotransmitter synthesis and increased depression risk; therapeutic intervention with L-methylfolate has been shown to be an effective adjuvant therapy for patients suffering from major depressive disorder [41, 42].

While pharmacogenetic testing in psychiatry is still emerging, there is a growing body of evidence demonstrating improved outcomes and cost effectiveness to support its utility and validity. Continued education and future research in the field are vital to its widespread acceptance. The knowledge and understanding of pharmacogenetics in psychiatry are continually growing, as is the application and utility into everyday clinical practice.

## 5. Clinician/Patient Perspectives

An important factor for the widespread adoption of genetic testing in psychiatry is clinician and patient acceptance. A study which utilized a random sample of US psychiatrists suggested that clinicians would be very open and welcome to pharmacogenetic testing in clinical practice [43]. Eighty-two percent of clinicians surveyed believed that testing to predict serious adverse effects would be somewhat or extremely useful, and 73% believe that testing to determine optimum dosages would also be somewhat or extremely useful [43]. This study also found that 80% of clinicians thought their patients would benefit from genetic testing, and 60% thought it would change the way psychiatry was practiced [43]. A separate study found that physicians in the psychiatric departments of three different academic institutions endorsed the use of pharmacogenetic testing and found it to be most useful in cases of treatment resistant depression and medication intolerance [27]. Most recently, 910 undergraduate medical students were surveyed regarding their views on genetic testing. Ninety percent of respondents indicated that if a genetic variant could help predict medication response or side effect risk, genetic testing should be utilized [44].

Several studies also examined patient perceptions to genetic testing. One study from UCLA found that cancer patients overwhelmingly (98.98%) would elect to receive predictive genetic testing at time of treatment even if no further treatment was available [45]. The study also found that results of genetic testing had no negative impact on the patients' quality of life or emotional well-being [45]. Seventy-eight to 86% of chronically ill patients, surveyed in 2002 and 2004, think the development of genetic research is hopeful for the treatment of disease, 77–85% think that it will lead to positive medical progress, and 76–85% approve of using deoxyribonucleic acid (DNA) testing for early detection of disease [46]. These data indicate that patients are open to genetic testing and feel it will improve treatment of chronic illnesses like depression. Knowledge and insight of one's illness along with positive beliefs and expectations of treatment are essential to patient treatment adherence and outcomes [47, 48]. These data show there is positivity and openness for testing in clinical practice which will help to facilitate the adoption as a regular treatment option.

## 6. Conclusion

Pharmacogenetic testing in psychiatry is a newly evolving field and is rapidly gaining wide acceptance. Numerous articles and reviews, as well as a growing number of case studies, have been published showing the clinical utility and cost effectiveness of genetic testing for psychiatric patients. To further substantiate the utility of genetic testing in psychiatry large randomized controlled trials are needed. The prevalence and burden of depression are predicted to continually grow [1]; the identification of effective treatment strategies will be instrumental to reduce patient burden as well as the economic consequences of mental disorders. Pharmacogenetic tests have the potential to change the way psychiatry and medicine as a whole are practiced. Genetic testing helps patients and clinicians answer the difficult questions regarding treatment failures and adverse events by helping to unravel the complexities of mental illness.

## 7. Future Perspective

In addition to the growing body of evidence on the utility of genetic testing to aid in the treatment of psychiatric disorders, there is also growing literature and promise regarding the utility of other genomic markers. Some of these other markers include small sections of ribonucleic acid (RNA) called microRNA (miRNA) responsible for playing a role in gene regulation, and gene expression levels analyzed using messenger RNA (mRNA). An additional layer of gene regulation is controlled by epigenetic modification which could also impact how genes are expressed.

MicroRNAs are small sections of RNA which can regulate up to several hundred genes and dysregulation of certain miRNAs may play a role in psychiatric and neurological disorders [49]. One example was identified in a recent study in patients with bipolar mania [50]. Patients who had lower plasma levels of miRNA-134 tended to have more severe symptoms as well as poorer treatment response to medication, indicating its role as a potential biomarker for treatment response [50].

Epigenetic modifications and mRNA gene expression levels are closely related as epigenetic changes can impact levels of mRNA gene expression. Epigenetic modification is defined as heritable changes in gene activity and expression which occurs without variation to the DNA sequence. These changes include DNA methylation and histone modification. A good example of both epigenetic and mRNA expression variations related to psychiatric treatment response can be found when examining the gene, brain-derived neurotrophic factor (*BDNF*). This gene has long been thought to be involved in antidepressant treatment response via genetic variation, epigenetic modification, and varied mRNA expression. BDNF mRNA expression levels have been found in several meta-analyses to be significantly decreased among psychiatric patients, and effective antidepressant treatments increase BDNF serum mRNA levels [51–53]. These studies indicate that plasma or serum BDNF mRNA levels could be a good biomarker of treatment efficacy/response. In addition, recent studies have also shown differences in methylation at various sites in* BDNF* may also be an indicator of antidepressant treatment response; however, these studies are still preliminary [51, 54, 55].

In the years to come, it is appears that pharmacogenetic testing will become integrated into everyday psychiatric practice. There is also great promise for additional genomic markers to be researched and developed to add information to further improve patient care. Pharmacogenetic/genomic testing will become a valuable tool to help improve patient outcomes, lower healthcare costs, and increase patient medication adherence. Just as pharmacogenetic testing has revolutionized clinical practice in areas such as cancer treatment, it has the potential to do so in psychiatry.

## Figures and Tables

**Table 1:** Cost effectiveness of genetic testing.

Study reference number	Gene(s)	Total number of study subjects	Average cost/resource use
[17–19]	CYP2D6 (extreme metabolism)	353	Testing reduces costs by 28%; PM have longer hospital stays; PM or UM have $4,000–6,000 higher costs

[17]	CYP2C19 (extreme metabolism)	104	Testing reduces costs by 28%

[20]	CYP2D6, CYP2C19, SLC6A4, CYP2C9, CYP1A2, and 5HTR2A	91	Patient with risks had 69% more healthcare visits, 67% more general medical visits, 3-fold more medical absence days, and 4-fold more disability claims

[21]	The Genecept Assay	333	Testing reduces outpatient costs by 9.5% or $562 over 4 months
